# An emerging phylogenetic core of Archaea: phylogenies of transcription and translation machineries converge following addition of new genome sequences

**DOI:** 10.1186/1471-2148-5-36

**Published:** 2005-06-02

**Authors:** Céline Brochier, Patrick Forterre, Simonetta Gribaldo

**Affiliations:** 1 Laboratoire EGEE (Evolution, Génomique, Environnement) Université Aix-Marseille I, Centre Saint-Charles, Case 36, 3 Place Victor Hugo, 13331 Marseille, Cedex 3, France; 2 Unite Biologie Moléculaire du Gène chez les Extremophiles, Institut Pasteur, 25 rue du Dr. Roux, 75724 Paris Cedex 15, France; 3 Atelier de Bioinformatique, Université Paris 6, 12 rue Cuvier, Paris, France

## Abstract

**Background:**

The concept of a genomic core, defined as the set of genes ubiquitous in all genomes of a monophyletic group, has become crucial in comparative and evolutionary genomics. However, it is still a matter of debate whether lateral gene transfers (LGT) may affect the components of genomic cores, preventing their use to retrace species evolution. We have recently reconstructed the phylogeny of Archaea by using two large concatenated datasets of core proteins involved in translation and transcription, respectively. The resulting trees were largely congruent, showing that informational gene components of the archaeal genomic core belonging to two distinct molecular systems contain a coherent signal for archaeal phylogeny. However, some incongruence remained between the two phylogenies. This may be due either to undetected LGT and/or to a lack of sufficient phylogenetic signal in the datasets.

**Results:**

We present evidence strongly favoring of the latter hypothesis. In fact, we have updated our transcription and translation datasets with five new archaeal genomes for a total of 6384 and 2928 amino acid positions, respectively, and 25 taxa. This increase in taxonomic sampling led to the nearly complete convergence of the transcription-based and translation-based trees on a single phylogenetic pattern for archaeal evolution. In fact, only a single incongruence persisted between the two phylogenies. This concerned *Methanopyrus kandleri*, whose placement remained strongly biased in the transcription tree due to its above average evolutionary rates, and could not be counterbalanced due to the lack of availability of closely related and/or slower-evolving relatives.

**Conclusion:**

To our knowledge, this is the first report of evidence that the phylogenetic signal harbored by components of the archaeal translation apparatus is confirmed by additional markers belonging to a second molecular system (i.e. transcription). This rules out the risk of circularity when inferring species evolution by small subunit ribosomal RNA and ribosomal protein sequences, since it has been suggested that concerted LGT may affect these markers. Our results strongly support the existence of a core of proteins that has evolved mainly through vertical inheritance in Archaea, and carries a *bona fide *phylogenetic signal that can be used to retrace the evolutionary history of this domain. The identification and analysis of additional molecular markers not affected by LGT should continue defining the emerging picture of a genuine phylogenetic core for the third domain of life.

## Background

The discovery that Lateral Gene Transfers (LGT) play a major role in the evolution of prokaryotic organisms has raised concerns about the possibility of reconstructing species phylogenies [1]. Some biologists even believe that LGT have obscured the phylogenetic record to such an extent that the task may be in fact hopeless [1,2]. However, others have argued that the careful selection of a 'core' of genes that have been refractory to transfer may help solving (at least partly) this conundrum [3-6]. The genomic core concept, i.e. the set of homologous genes present in all -or most-genomes of a phylogenetically coherent group, has become crucial in comparative and evolutionary genomics [7]. Indeed, the identification of 'genomic cores' can provide crucial information on the composition of ancestral genomes [8,9], as well as on organisms evolution at various phylogenetic depths [6,10,11]. However, homology-based analyses to define core genes cannot discriminate between vertically transmitted components and horizontally exchanged ones (i.e. "cryptic orthologous replacements" [7]). Thus, it is still a matter of debate whether Lateral Gene Transfers (LGT) may affect the components of genomic cores, preventing their use to retrace species evolution. Nevertheless, the extent and nature of the horizontal component of genomic cores can be identified by molecular phylogeny. The use of conserved gene cores to retrace species evolution has mainly focused on the translation apparatus, since the ribosome appears to be one of the best conserved macromolecular machines in the living world. The concatenation of either bacterial and archaeal ribosomal protein sequences has produced global phylogenies that are roughly similar to those obtained with both small and large ribosomal subunit rRNA genes (16S and 23S rRNA) [4,5,11]. Moreover, careful individual analyses have indicated that ribosomal proteins have been apparently never exchanged between the three Life domains, and rarely between different lineages within domains [4,5,12,13]. However, it may be argued that concerted LGT involving rRNA and ribosomal protein genes, since they belong to the same macromolecular machinery, could escape detection in such analyses. Nevertheless, this hypothesis could be discarded if phylogenies based on additional sets of genomic core proteins belonging to other molecular machineries are congruent with those of the components of the translation apparatus.

**Figure 1:**
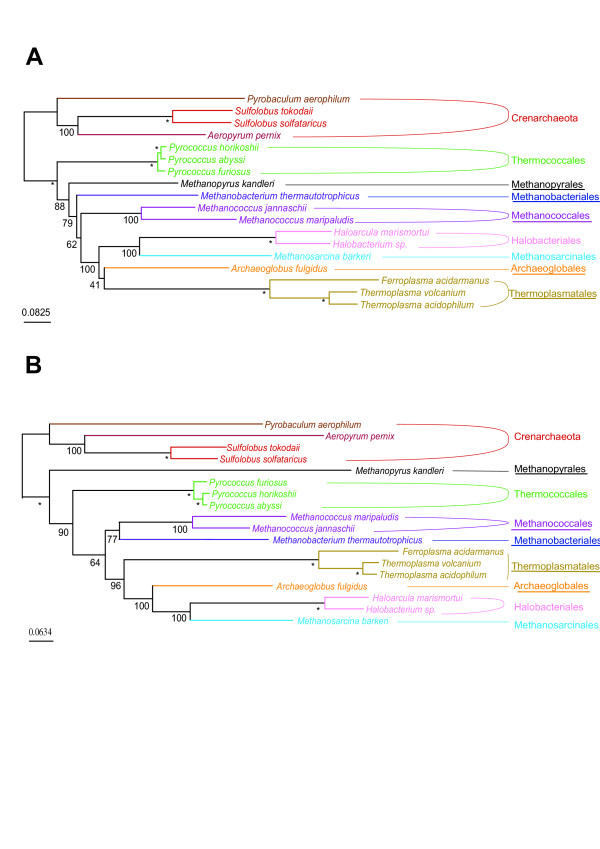
Unrooted Maximum Likelihood (ML) trees based on concatenation of ribosomal proteins (A) and RNA polymerase subunits and transcription factors (B). For clarity, the two datasets from Brochier et al. (2004) were scaled down to the same number of species by removing *Methanosarcina mazei *and *Methanosarcina acetivorans *from the transcription dataset. Numbers at nodes are bootstrap values (BV). The scale bars represent the number of changes per position for a unit branch length. Trees were produced by exhaustive searches performed by PROTML. Branch lengths and likelihood values were calculated by TREE-PUZZLE (JJT model including a Γ-correction (8 categories of sites)). Numbers at nodes are bootstrap values computed with PUZZLEBOOT from 1000 replications. Asterisks indicate constrained nodes (supported by BV = 100% in preliminary NJ and heuristic ML analyses). The names of groups showing incongruent positions between the two trees are underlined.

Accordingly, we have recently performed an in-depth analysis of proteins involved in transcription and translation from Archaea [11]. Individual phylogenies of these proteins confirmed that the components of these informational molecular machineries are little affected by LGT in the archaeal domain, and permitted the assembly of two large concatenated datasets of likely vertically-transmitted genes to reconstruct the phylogeny of the third domain of life [11]. The trees based on the 'translation' dataset (53 ribosomal proteins, Figure 1A) and the 'transcription' dataset (11 RNA polymerase subunits and 3 transcription factors, Figure 1B) were globally congruent, suggesting that the two informational systems contain a coherent phylogenetic signal for the archaeal phylogeny [11]. However, a number of incongruent nodes remained between the two trees (Figure 1A and 1B). First, the hyperthermophilic methanogen *Methanopyrus kandleri *was close to other methanogens in the translation tree (Figure 1A), whereas it emerged with a strong statistical confidence at the base of the euryarchaeal phylum in the transcription tree (Bootstrap Value BV = 90%, Figure 1B). A second incongruence concerned the position of the euryarchaeon *Archaeoglobus fulgidus*, since this archaeon was grouped, albeit with weak support (BV = 41%), with Thermoplasmatales in the translation tree (Figure 1A), whereas in the transcription tree it was strongly placed as sister group to the clade composed of Methanosarcinales and Halobacteriales (BV = 100%) (Figure 1B). Finally, although in both phylogenies Methanobacteriales and Methanococcales were located in-between Thermococcales and a large clade comprising Thermoplasmatales, *Archaeoglobus*, Methanosarcinales and Halobacteriales, they were paraphyletic in the translation tree (Figure 1A) whereas they were monophyletic in the transcription tree (Figure 1B).

In the case of *M. kandleri*, we suggested that the discrepancy between the translation and transcription trees was likely due to the very fast evolutionary rate of its RNA polymerase subunits (reflected by the very long branch of *M. kandleri *in the transcription tree, Figure 1B). Such an accelerated rate of evolution may be due to the lack in this archaeon of the critical transcription factor TFS [11,14]. A Long Branch Attraction (LBA) artefact [15] between the very long branch of *M. kandleri *and the outgroup (i.e. Crenarchaeota) may thus be responsible for the basal position of this methanogen in the transcription tree. In contrast, the incongruence between the two trees in the position of *A. fulgidus*, and in those of Methanobacteriales and Methanococcales may be either due to undetected LGT, and/or result from an insufficient phylogenetic signal in the two protein datasets. Only in the latter case should an increased taxonomic sampling help resolving this incongruence, whereas if LGTs are responsible, the addition of more taxa should not increase resolution and will possibly add more confusion. The recent sequencing of several new genomes from Euryarchaeota now permits tackling these two alternatives.

## Results and discussion

We have updated our previous datasets of the components of the translation and transcription machineries [11] to include a total of 25 Archaea. In particular, we included the psychrophilic methanogen *Methanogenium frigidum *[16] and the mesophilic methanogen *Methanococcoides burtonii *[16]-two lineages belonging to Methanomicrobiales [17] and Methanosarcinales [18], respectively. We also included the halophile *Haloferax volcanii *[19], the Thermococcale *Thermococcus gammatolerans *(Yvan Zivanovic and Fabrice Confalonieri, personal communication), and *Nanoarchaeum equitans*, a highly divergent archaeon that has been suggested as the representative of a new archaeal phylum, the Nanoarchaeota [20-22]. As in our previous studies [5,11] we did not include any eukaryotic outgroup in order to limit biases due to LBA.

As described previously [11], separated phylogenetic analyses were performed on each of these new datasets in order to identify and remove potential lateral gene transfer (LGT) events (data not shown). Despite the fact that most relationships were largely unresolved in several trees due to the small size of most proteins, we checked for any possible strongly supported departure from undisputed nodes in the archaeal phylogeny, such as the clades of Thermoplasmatales, Halobacteriales, Sulfolobales, Thermococcales, Methanosarcinales and Methanococcales. Following the addition of novel taxa, no new clear-cut case of LGT could be observed with respect to these nodes, confirming that transfers are indeed very rare for these markers[5,11]. A few proteins gave an instable placement for *Nanoarchaeum equitans*. However, since the position of this taxon in the archaeal phylogeny has not yet been firmly tested, we did not judge these proteins as clear-cut cases of LGT. The 53 ribosomal proteins and the 14 proteins involved in transcription were thus concatenated into two large 'translation' and 'transcription' datasets, whose sizes were 6384 and 2928 amino acid positions, respectively.

**Figure 2:**
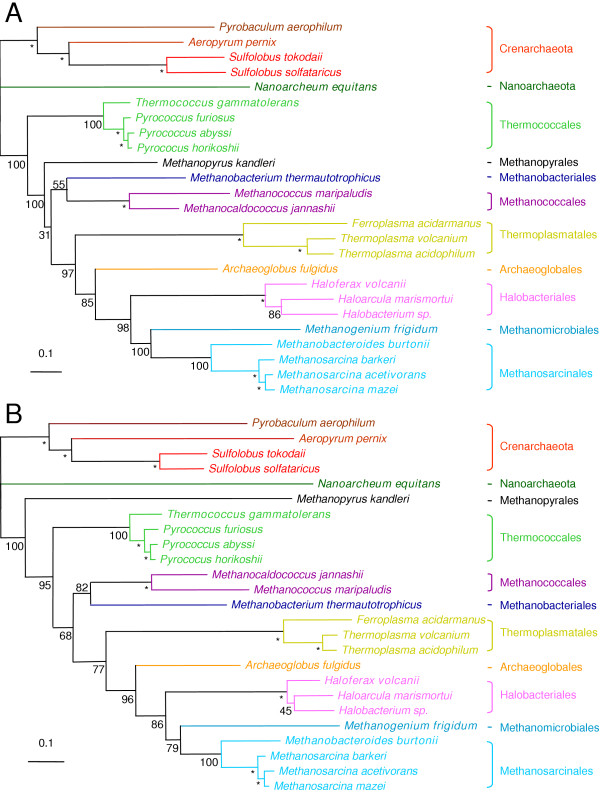
Unrooted ML trees for the updated translation dataset (A) and transcription dataset (B). For details and tree computation see legend to Figure 1. The five new taxa are indicated in bold.

Exhaustive Maximum Likelihood (ML) searches were performed on the two updated translation and transcription fusion datasets, with a few constraints given to undisputable nodes (i.e. supported by BV = 100% in preliminary Neighbor Joining and ML heuristic analyses (not shown)). The best ML topologies for the translation and transcription datasets are presented in Figure 2A and 2B, respectively. Topologies not significantly less likely than the ones presented in Figure 2 differed by minor rearrangements on nodes that are feebly supported by bootstrap, such as the branching order within halobacteriales in the transcription tree, or the grouping of methanopyrus with methanococcales/methanobacteriales in the translation tree (not shown).

Interestingly, the addition of five new archaeal taxa led to convergence of the transcription and translation trees on a coherent phylogenetic pattern, to the exclusion of the position of *M. kandleri*-still emerging after Thermococcales in the translation tree (Figure 2A), but recovered as a very long branch at the base of the euryarchaea in the transcription tree (Figure 2B)-(the same trees were obtained when removing *M. kandleri *from the datasets, data not shown). This only incoherence between the two phylogenies is most likely due to the fact that the LBA artefact affecting the position of *M. kandleri *in the transcription tree persisted even after increase in taxonomic sampling, due to unavailability of closely related and slower evolving species. *Nanoarchaeum equitans *emerged as a separate branch distinct from those leading to Crenarchaeota and Euryarchaeota domains, in both translation and transcription trees (Figure 2A and 2B), supported by strong bootstrap values (BV = 100%). This position is congruent with previous results based on ribosomal proteins concatenation [22]. *T. gammatolerans *branched off of at the base of Thermococcales, that were confirmed as the first emerging euryarchaeal phylum, as in our previous studies [5,11]. Interesting, Methanobacteriales and Methanococcales formed now a monophyletic group in both translation and transcription trees (BV = 55% and 82%, Figure 2A and 2B, respectively). This suggests that the paraphyly of these groups observed in our previous translation tree (Figure 1A) was likely incorrect due to a lack of phylogenetic signal rather than to a LGT bias. The Methanobacteriales/Methanococcales monophyletic group is sister to a large cluster including both methanogenic and non-methanogenic species: *A. fulgidus*, the three Thermoplasmatales, the three Halobacteriales and the five Methanomicrobia (Methanomicrobiales and Methanosarcinales) (BV = 97% and BV = 77%, Figure 2A and 2B, respectively). This supports the hypothesis of an ancient origin of methanogenesis in Archaea followed by subsequent loss in some lineages (*A. fulgidus*, Thermoplasmatales and Halobacteriales). Moreover, the position of *A. fulgidus*, while left uncertain in our previous analyses (i.e. either sister-group of Thermoplasmatales in the translation tree, or of Halobacteriales/Methanomicrobia in the transcription tree, Figure 1A and 1B, respectively) was now robustly indicated as sister to Methanomicrobiales, Methanosarcinales and Halobacteriales in both translation and transcription trees (BP = 85% and 96%, respectively, Figure 1A and 1B). The strong placement of *A. fulgidus *in our updated translation tree is likely due the stabilisation of the node following addition of new taxa. This result further supports the hypothesis of a late and independent emergence of aerobic respiration in Euryarchaeotes (Halobacteriales), possibly via the recruitment of bacterial genes. Finally, both translation and transcription trees confidently grouped *M. burtonii *and *M. frigidum *with the three Methanosarcina (BV = 100% and BV = 100%, Figure 2A and BV = 100% and BV = 79%, Figure 2B, respectively) within the Methanomicrobia group. The very close relationship between *M. burtonii *and the three Methanosarcinales constitutes a novel phylogenetic argument justifying its inclusion in the order Methanosarcinales, at present based only on 16S rRNA phylogeny [18].

## Conclusion

The congruence we obtained between the archaeal phylogenies based on the components of the translation and transcription machineries strongly supports the existence of a core of genes that evolved mainly through vertical inheritance in Archaea, and carry a *bona fide *phylogenetic signal that can be used to infer the phylogeny of this domain. Our results confirm also that the addition of new taxa strongly improves phylogenetic inference, and support the idea that evolutionary considerations should be included in the choice of new genomes to be sequenced. However, our conclusions should not be considered as the "last word" on the subject. For example, the misplacement of *M. kandleri *at the base of Euryarchaea in the transcription tree was not cured by the increase in taxonomic sampling. The inclusion of sequences from slower evolving and close relatives, when they will be available, may help resolving this bias. Similarly, the very long branch displayed by *N. equitans *(Figure 2A and 2B) suggests that its placement as a separate branch distinct from that leading to Euryarchaeota and Crenarchaeota, although congruent between the transcription and translation trees, should be taken with caution due to the risk of an LBA artefact. The analysis of the components of additional molecular systems and the inclusion of more taxa may eventually lead to a confident placement for these two interesting species in the archaeal phylogeny.

Finally, our results make us confident that the construction of a phylogeny that retraces the vertical history of the archaeal domain is a feasible task. The identification and analysis of additional molecular markers not affected by LGT on large phylogenetic scales and their phylogenetic analysis by approaches that minimise reconstruction artefacts should continue defining the emerging picture of a genuine phylogenetic core for the Archaea. The application of a similar strategy to the bacterial and eukaryal domains could also lead to a *bona fide *reconstruction of their respective evolutionary histories.

## Methods

In order to update the datasets of our previous analysis [11], we included the two methanogens *Methanogenium frigidum *[16] and *Methanococcoides burtonii *[16], the halophile *Haloferax volcanii *[19], the Thermococcale *Thermococcus gammatolerans *(Yvan Zivanovic and Fabrice Confalonieri, personal communication), and *Nanoarchaeum equitans*. In addition, the sequences of the two Methanosarcinales *Methanosarcina mazei *and *Methanosarcina acetivorans *were added to the ribosomal dataset. Sequences were retrieved by TBLASTN at genome sequencing web sites for *H. volcanii *, *M. burtonii *and *M. frigidum *, or using BLASTP in NCBI for *N. equitans*, *M. acetivorans *and *M. mazei *[23]. For each dataset, novel sequences were manually added to previous alignments by using the ED program of the MUST package [24]. Regions were the alignment was ambiguous were removed from the each dataset.

Trees were computed by a number of different approaches. Neighbor-Joining (NJ) trees were calculated by the NEIGHBOR program of the PHYLIP package [25], using Maximum Likelihood (ML) distance matrices (JTT model including a Γ-correction) computed by TREE-PUZZLE 5.1 [26]. Heuristic ML trees were computed using PHYML with the JTT model including a Γ-correction [27]. Exhaustive tree topology searches with limited constraints were performed using PROTML of the MOLPHY package [28]. The likelihoods and branch lengths of ML topologies were performed by TREE-PUZZLE (JTT model including a Γ-correction). For exhaustive ML searches, constraints (asterisks in Figures 1 and 2) were given to undisputable nodes (supported by BV = 100%), based on preliminary NJ and ML heuristic analyses (not shown).

The SEQBOOT program of the PHYLIP package [25] was used for the generation of bootstrapped datasets, and PUZZLEBOOT [29] and CONSENSE in the PHYLIP package [25] were used for bootstrap value calculations on 1000 replications and consensus tree reconstructions, respectively.

Datasets and their corresponding phylogenies are available on request from CB.

## Authors' contributions

CB carried out the analyses. CB, PF and SG conceived the study and drafted the manuscript. All authors read and approved the final manuscript.
